# Efficacy and Safety of Platelet-Rich Plasma in Melasma: A Systematic Review

**DOI:** 10.7759/cureus.63746

**Published:** 2024-07-03

**Authors:** Nujud M Alshammari, Zaid Z Almustafa, Hassan N AlBaqshi, Zahra Abu Jawhar

**Affiliations:** 1 Dermatology, Eastern Health Cluster, Dammam, SAU; 2 Dermatology, Qatif Health Network, Eastern Health Cluster, Qatif, SAU; 3 Dermatology, Dammam Health Network, Dammam, SAU

**Keywords:** hydroquinone, melasma, dermatology, efficacy, platelets-rich plasma

## Abstract

Melasma is a prevalent dermatological challenge with limited therapeutic interventions. Platelet-rich plasma (PRP) has been increasingly explored for its potential benefits in various dermatological conditions. This study aimed to systematically review the efficacy and safety of PRP in the treatment of melasma. A comprehensive search in line with the Preferred Reporting Items for Systematic Reviews and Meta-Analyses (PRISMA) guidelines was executed in January 2024 using PubMed, focusing on studies investigating the efficacy and safety of PRP in melasma. Criteria for inclusion were clinical trials and controlled studies examining PRP’s role in melasma treatment, while exclusions covered reviews, non-English articles, and studies older than 10 years, among others. Eight studies were included, with the majority targeting female participants. The research displayed consistent positive outcomes, whether PRP was used alone or synergistically with treatments like hydroquinone and tranexamic acid. However, positive studies with the combination of PRP and other drugs will not provide the actual safety and efficacy data of PRP. The combined treatment approaches often showed enhanced results. Satisfaction rates among patients and reductions in the melasma area and severity index (MASI) scores were common findings across the studies, emphasizing the potential of PRP in melasma management. In conclusion, PRP emerges as a promising therapeutic intervention for melasma. Whether as a standalone treatment or combined with established methods, PRP presents significant potential in melasma’s clinical management, warranting further expansive trials to substantiate its long-term efficacy and safety.

## Introduction and background

Introduction

Melasma is a common, acquired hypermelanosis characterized by symmetrical, blotchy, brownish facial pigmentation. It primarily affects women, especially those residing in areas with intense sun exposure, though men can also be affected [1]. Though not life-threatening, melasma can result in substantial emotional and psychological distress due to its prominent appearance on the face and the potential for recalcitrance or recurrence [2].

Multiple factors contribute to melasma’s pathogenesis, including genetic predisposition, ultraviolet (UV) radiation, hormonal fluctuations (like those experienced during pregnancy or with oral contraceptive use), certain medications, and inflammation [3]. While a range of treatment options exists, including topical lightening agents, chemical peels, and laser treatments, the chronic nature of melasma often leads to incomplete responses and recurrence upon cessation of therapy or continued sun exposure [4].

Platelet-rich plasma (PRP) has emerged as a promising therapeutic agent in various medical and aesthetic applications. PRP is an autologous concentration of human platelets in a small volume of plasma, containing various growth factors and cytokines that can promote tissue regeneration, angiogenesis, and collagen production [5]. Because of its regenerative properties, PRP has been used in wound healing, orthopedic surgeries, hair restoration, and aesthetic skin rejuvenation [6].

Melasma’s management is multifaceted and requires a combination of preventive, therapeutic, and maintenance strategies to achieve satisfactory results. A cornerstone of melasma treatment involves protection against UV radiation, as sun exposure is a well-established trigger for melasma exacerbation. Regular use of broad-spectrum sunscreens with a sun protection factor (SPF) of at least 30 is recommended, along with physical measures like wearing wide-brimmed hats and avoiding peak sunlight hours [7].

Topical therapies remain the mainstay of treatment for melasma. The most commonly prescribed agent is hydroquinone, which acts as a tyrosinase inhibitor, preventing melanin synthesis. Hydroquinone, available in concentrations ranging from 2% to 4%, is often used in combination with other agents, such as tretinoin and corticosteroids, to enhance efficacy and minimize irritation [8]. Apart from hydroquinone, other topical agents like azelaic acid, kojic acid, and glycolic acid have also demonstrated efficacy in reducing melasma severity [9].

Chemical peels, employing agents like glycolic acid, trichloroacetic acid (TCA), and salicylic acid, serve as another treatment modality. These peels work by exfoliating the upper layers of the skin, thereby accelerating the removal of melanin. It is crucial, however, to select the appropriate peel type and concentration based on the patient’s skin type to minimize potential side effects, such as irritation or post-inflammatory hyperpigmentation [10].

In recent years, oral agents, such as tranexamic acid (TXA), and antioxidants like vitamins C and E, have garnered attention in melasma management. TXA, an antifibrinolytic agent, has shown efficacy in reducing melasma when administered orally, although it is generally reserved for cases resistant to traditional therapies due to concerns about potential side effects [11].

Given the multifactorial nature of melasma, treatments addressing the inflammatory and degenerative aspects can potentially be beneficial. PRP, with its anti-inflammatory and tissue-regenerating properties, offers a novel avenue for melasma treatment. However, the literature on PRP’s efficacy and safety in melasma is scattered, with varying study designs, outcomes, and conclusions. This necessitates a systematic review to collate the evidence and provide clinicians with a clear understanding of PRP’s role in melasma management.

This systematic review aims to critically evaluate the available literature on the efficacy and safety of PRP as a therapeutic intervention for melasma, synthesizing evidence to guide clinical decisions and identify gaps in the existing research.

## Review

Methodology

The PRISMA (Preferred Reporting Items for Systematic Reviews and Meta-Analyses) guidelines were followed for this systematic review.

Study design and duration

This was a systematic review conducted in January 2024.

Search strategy

To retrieve the relevant research, a thorough search was conducted across major databases, Using PubMed Mainly as a search engine for studies. We only searched in English. The following keywords were converted into PubMed Mesh terms and used to find studies that were related; “Platelets-Rich Plasma,” “Efficacy,” “Dermatology,” and “Melasma.” The Boolean operators “OR” and “AND” matched the required keywords. Among the search results were publications in full English language, freely available articles, and human trials.

Selection criteria

Inclusion Criteria

Studies that investigate the efficacy of PRP in melasma, studies that study the safety of PRP in melasma treatment, and clinical trials and controlled clinical studies were included.

Exclusion Criteria

Systemic reviews, studies that focused on other dermatological diseases other than melasma, article reviews, meta-analysis, studies older than 10 years, case reports, letters to the editors, and replies to conflicts and studies in non-English language were excluded.

Data extraction

Duplicates in the search strategy output were found using Rayyan (QCRI) [12]. To determine the titles’ and abstract relevance, the researchers used a set of inclusion/exclusion criteria to filter the combined search results. The reviewers carefully read each paper that matches the requirements for inclusion. The authors provided other methods of resolving disputes with some thought. The authors extracted data about the study titles, authors, study year, country, participants, gender, diagnostic tool, main outcomes, and conclusion.

Strategy for data synthesis

Summary tables were created using information from pertinent research to give a qualitative overview of the results and study components. Following data extraction for the systematic review, the most effective strategy for utilizing data from the included study articles was selected.

Risk of bias assessment

Using the ROBINS-I risk of bias assessment approach for non-randomized trials of therapies, the included studies’ quality was assessed [13]. The seven themes that were assessed were confounding, participant selection for the study, classification of interventions, deviations from intended interventions, missing data, assessment of outcomes, and choosing of the reported result.

Results

Search Results

**Figure 1:**
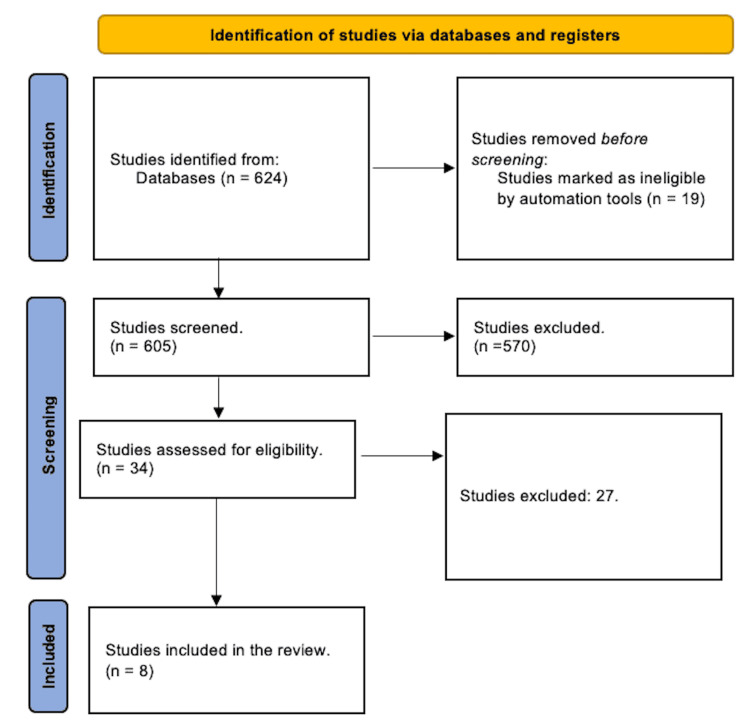
PRISMA flowchart summarizes the study selection process. PRISMA: Preferred Reporting Items for Systematic Reviews and Meta-Analyses

A total of 605 study articles resulted from the systematic search, and 19 were automatically removed for being older than 10 years. Title and abstract screening were conducted on 605 studies, and 570 studies were excluded. Finally, 35 studies were screened for assessment; 27 were excluded for either having inappropriate study methodology or results. Eight eligible study articles were included in this systematic review. A summary of the study selection process is presented in Figure 1.

Characteristics of the included studies

A comprehensive overview of participants from eight distinct studies is provided in Table 1, amounting to a total participant count of 313 from references [14-21]. These studies are spread across different countries: Egypt, Thailand, India, Pakistan, and China.

**Table 1:** Sociodemographic characteristics of the included participants

Study	Study design	Location	Participants	Age range (mean) in years	Males (%)
Hofny et al. 2019 [14]	Clinical trial	Egypt	23	-	-
Sirithanabadeekul et al. 2020 [15]	Randomized, single-blinded prospective trial	Thailand	10	46.2	0%
Tuknayat et al. 2021 [16]	Open-labeled prospective trial	India	40	-	10%
Bikash et al. 2022 [17]	Randomized trial	India	30	-	-
Mumtaz et al. 2021 [18]	Non-randomized controlled trial	Pakistan	64	24 ± 9	54.69%
Gamea et al. 2022 [19]	Randomized trial	Egypt	40	-	0%
Zhang et al. (2022) [20]	Retrospective analysis	China	80	28.92 ± 4.7	12.5%
Gharib et al. (2021) [21]	Single-center clinical trial	Egypt	26	35.46 ± 5.09	0%

When it comes to gender representation, the overwhelming studies focused on female patients. Sirithanabadeekul et al. 2020 [15] reported no male participants. Similarly, Gamea et al. 2022 [19] and Gharib et al. 2021 [21] both documented studies with a 0% male population. On the other hand, Mumtaz et al. 2021 [18] was the only study that had a considerably higher male representation, with 54.69% of their study participants being male. Tuknayat et al. 2021 [16] mentioned a male percentage of 10%, while Zhang et al. 2022 [20] recorded 12.5% of their participants as male. Hofny et al. 2019 [14] and Bikash et al. 2022 [17] did not provide data on the gender distribution in their studies.

In terms of the study designs, except for one study, all studies were clinical trials, with varying methodologies, such as randomized, single-blinded prospective trials, open-labeled prospective trials, and non-randomized controlled trials. A distinct approach was seen by Zhang et al. 2022 [20], who utilized a retrospective analysis for previously treated patients in their hospital for their study. With regard to the age range, not all studies provided age data. However, of those that did, Mumtaz et al. 2021 [18] highlighted a mean age of 24 ± 9 years, Zhang et al. 2022 [20] presented an average age of 28.92 ± 4.7 years, and Gharib et al. 2021 [21] documented participants with a mean age of 35.46 ± 5.09 years. Sirithanabadeekul et al. 2020 [15] was the only study to specifically focus on older individuals, with a mean age of 46.2 years. In summation, based on the studies that detailed age specifics, it's evident that the majority of the research predominantly focused on adults, with a mean age trending toward the late twenties and early thirties.

Clinical outcomes of these studies are provided in Table 2. The effectiveness of PRP in the treatment of melasma has been extensively studied. Hofny et al. 2019 [14] explored PRP’s efficiency through two delivery methods, namely microneedling with a dermapen and microinjections with mesoneedles. Their findings revealed that both methods significantly reduced the melasma area and severity index (MASI) and modified MASI (mMASI) scores, indicating that PRP is a potent treatment option. Similarly, Tuknayat et al. 2021 [16] confirmed PRP’s efficacy as a standalone therapy, observing a 54.5% average reduction in mMASI scores over six months, with over 90% of patients expressing satisfaction with the results and no cases of relapse.

**Table 2:** Clinical characteristics and outcomes of the included studies TXA: tranexamic acid; PRP: platelet-rich plasma; VEGF: vascular endothelial growth factor; MSH: melanocyte-stimulating hormone; ET1: endothelin-1

Study	Methodology & Objective	PRP Preparation	Results	Outcomes/Conclusion
Hofny et al. 2019 [14]	The study assessed the effectiveness of PRP in treating melasma using two delivery methods: microneedling with dermapen and microinjections with mesoneedles.	10 mL of blood was collected on anticoagulant and centrifuged to separate it into three layers: red blood cells (RBCs), platelet-poor plasma (PPP), and a “buffy coat” layer. The RBCs layer was discarded, and the PPP layer was discarded. A second centrifugation step was performed, focusing on the “buffy coat” layer with a small amount of PPP 6. Platelet activation was achieved by adding 3% calcium chloride to the 1.5 mL of PRP obtained after centrifugation.	After PRP treatment, the melasma area and severity index (MASI) and modified MASI (mMASI) scores significantly reduced. Both delivery methods were effective, with no significant difference between them.	PRP is a valuable treatment option for melasma, with both microneedling and microinjections being effective delivery methods.
Sirithanabadeekul et al. 2020 [15]	This study aimed to evaluate the effectiveness of PRP treatment. Over four sessions, 10 female patients received intradermal PRP injections on one side of their face and normal saline on the other.	The anticoagulant citrate dextrose A and 13.5 mL of blood were drawn into a 20-mL syringe using a butterfly cannula, shaken for 15 seconds, and then transferred to the YCELLBIO Kit®. The anticoagulant-blood mixture was then transferred to the device and centrifuged at 3200 revolutions per minute for 4 minutes at room temperature. The buffy coat and plasma at the device's neck were extracted using a 3mL syringe and an 18-gauge needle before being removed slowly.	Significant improvements in the PRP condition were observed regarding the mMASI score, Antera® 3D-assessed melanin levels, and patient satisfaction. However, Mexameter®-assessed erythema and melanin indices displayed no significant differences between the PRP and control conditions.	PRP treatment showcased promising results in melasma management, suggesting its potential as an alternative or complementary treatment.
Tuknayat et al. 2021 [16]	This study evaluated the effectiveness of PRP as a standalone treatment for melasma, administering intralesional PRP to 40 patients once a month for three sessions.	PRP was prepared by manual double centrifugation using 10 mL of patients’ venous blood in a refrigerated centrifuge at 4°C. The first centrifugation was at 1600 rpm for 10 minutes, followed by extraction of the buffy coat layer. The second centrifugation was at 4000 rpm for another 10 minutes. A pellet of platelets was re-suspended into one-third of the remaining plasma. Multiple injections of PRP were given to all melasma lesional areas in a dose of 0.1 mL/cm^2^ after topical anesthesia. Treatment sessions included three monthly sessions and a follow-up for 6 months.	The study observed an average 54.5% reduction in the mMASI score at the end of six months. Over 90% of the patients self-evaluated their improvement positively, and there were no cases of relapse.	PRP demonstrated significant efficacy as a standalone therapy for melasma, with most patients pleased with the results and no significant side effects noted.
Bikash et al. 2022 [17]	The study aimed to compare the effectiveness of PRP therapy combined with hydroquinone against hydroquinone alone in treating melasma. Thirty patients were randomized to receive PRP microinjections on one side of their face and normal saline on the other.	PRP process not included in the article.	The PRP + HQ group witnessed significant improvements in MASI scores with 40% having 51% to 75% improvement, compared to only 3.3% in the HQ alone group. Furthermore, 53.3% in the PRP + HQ group reported a good response, versus 27% in the HQ group.	Combining PRP therapy with hydroquinone demonstrates enhanced efficacy in melasma treatment compared to using hydroquinone alone.
Mumtaz et al. 2021 [18]	The study aimed to compare the effectiveness of intradermal PRP versus intradermal TXA for treating melasma. over 24 weeks, treatments were given every 4 weeks.	15-20 mL of blood was taken in a tube with sodium citrate anticoagulant, and PRP was obtained manually using a centrifuge machine. The upper 2/3rd was discarded, while the lower 1/3rd was PRP. Calcium chloride was added to activate the platelets before injection. PRP was injected intra-dermally through a 30G needle in each cm^2^ melasma. In group B, insulin syringes were used with a volume of 1 mL, containing 0.04 mL of TXA and 4 mg of normal saline. Injection of TXA was injected intradermally through a 30G needle in each cm^2^ melasma. Treatment was offered every 4 weeks for 12 weeks, with a final outcome at 24 weeks.	Out of 64 participants, half were treated with PRP (Group A) and half with TXA (Group B). MASI scores showed better improvement in Group A compared to Group B, especially evident at the 4-week, 12-week, and 24-week check-ins.	Intradermal PRP has a more significant positive effect on melasma treatment than intradermal TXA.
Gamea et al. 2022 [19]	The study aimed to evaluate the efficacy of 5% topical TXA in a liposome base alone versus its combination with intradermal PRP in treating melasma. The group was that was getting PRP injections, injected every three weeks, and both were treated for 12 weeks.	PRP was prepared by withdrawing venous blood from a patient using a butterfly cannula and a vacuum tube with sodium citrate as an anticoagulant. The plasma was centrifuged at 2000 rpm for 3 minutes and 5000 rpm for 5 minutes, and 0.1 mL of calcium chloride was added to activate platelets.	Both groups observed significant improvement in their mMASI scores post-treatment, but group B (those receiving PRP) had notably better results and patient satisfaction. PRP side effects were mild, and TXA was well received.	The use of 5% topical TXA in a liposome base is deemed both safe and effective for melasma treatment, and the results can be enhanced further with PRP injections.
Zhang et al. (2022) [20]	The research aimed to study the effect of combining PRP with oral TXA in treating melasma, focusing on the serum levels of VEGF, ET-1, and MSH.	The patient’s venous blood was drawn, centrifuged, and suspended to form PRP. The instrument was adjusted to very fast, and a fixed dose of 0.0179 to 0.0208 mL was given. The PRP was then injected under the melasma lesion. This is a whole face injection treatment, once a month, three times. The equipment used included the Regen ACRC (Regen PRP, Regenlab, Switzerland), centrifuge (Micro 17; Thermo Fisher Scientific), and water light injection instrument.	The combined PRP and TXA treatment was more effective (90.48%) than TXA alone (73.68%). The study group experienced more pronounced changes in serum levels of the specified markers. The recurrence rate was lower in the study group 6 months post-treatment.	PRP combined with oral TXA offers a superior treatment outcome for melasma compared to TXA alone, impacting specific serum levels and reducing disease recurrence.
Gharib et al. (2021) [21]	The study aimed to compare the effects of microneedling followed by PRP and microneedling followed by TXA on patients with melasma.	The study involved collecting autologous whole blood into tubes containing acid citrate dextrose and centrifuging at 1,500 rpm for 10 minutes to retain platelet count. The PRP was then centrifuged at 3,700 rpm for 10 minutes at 22°C to obtain a platelet count 4.5 times higher than the baseline. PPP was used to resuspend platelets and calcium gluconate was added as an activator. TXA was prepared as a 5-mL ampoule containing 500 mg of the active ingredient, and then topically applied.	Using the MASI scoring system, no significant differences were noted at the start of the study, but the second and third sessions showed significant differences. Both treatments improved melasma, but the response differed between the groups over the sessions.	Microneedling combined with PRP is more effective than microneedling with TXA in treating melasma.

Sirithanabadeekul et al. 2020 [15] and Gamea et al. 2022 [19] approached PRP’s effectiveness from a comparison standpoint. Sirithanabadeekul et al.’s research pitted PRP against normal saline, discovering that PRP outperformed the saline control in terms of mMASI score reductions, melanin levels, and patient satisfaction. Gamea et al.’s study evaluated the combined efficacy of 5% topical TXA and PRP, revealing that the PRP-enhanced group displayed superior results and heightened patient satisfaction.

Discussion

This comprehensive review aggregates data from eight studies spanning Egypt, Thailand, India, Pakistan, and China, totaling 313 participants [14-21]. Predominantly, these studies targeted female subjects, with no male representation in studies by Sirithanabadeekul et al. 2020 [15], Gamea et al. 2022 [19], and Gharib et al. 2021 [21]. However, Mumtaz et al.’s study (2021) [18] was an exception, showing a high male percentage at 54.69%. Concerning study methodologies, most were clinical trials of varied designs, except Zhang et al.’s (2022) [20], which employed a retrospective approach. Regarding age, while not consistently provided, available data indicates a focus on adults, particularly in their late twenties to early thirties.

The exploration into the therapeutic potential of PRP in the realm of melasma treatment has offered noteworthy insights. Hofny et al. 2019 [14] pioneered this research trajectory by scrutinizing the efficacy of PRP and introducing a comparative analysis of its delivery mechanisms-microneedling versus microinjections. Their results elucidated a marked decline in MASI and mMASI scores post-PRP intervention, reaffirming its therapeutic promise. Intriguingly, the study broke new ground by being the first to juxtapose the efficiency of these two delivery methods, arriving at the conclusion that both are comparably efficacious.

Sirithanabadeekul et al. 2020 [15], in a meticulous randomized, placebo-controlled trial-remarkably the first of its kind focusing on PRP for melasma-delved deeper into this therapeutic avenue. The study design encompassed a comparative analysis between PRP treatment and a saline control, offering robust findings. Not only did the PRP cohort manifest a substantial reduction in melanin and wrinkle levels, but the mMASI scores also registered a noteworthy dip. What buttresses the potential of PRP here is the escalating patient satisfaction over the study duration. Although certain parameters like the melanin index did not demonstrate statistical significance, the overarching outcomes favor the candidacy of PRP as a prospective melasma therapy. Nevertheless, the study posits a need for expansive trials to validate PRP’s long-term safety and efficacy.

Subsequently, Tuknayat et al. 2021 [16] took a singular focus on PRP as a standalone therapeutic agent for melasma. With a predominantly female cohort possessing Fitzpatrick IV and V skin types, the results were compelling. An overwhelming 90% of the participants resonated with positive outcomes, registering a stark 54.5% average decrement in their mMASI scores. The absence of significant side effects and relapses, coupled with the high satisfaction rates, further accentuated PRP’s potential as an autonomous treatment regime for melasma.

The investigation by Bikash et al. 2022 [17] adopted a synergistic approach by evaluating the amalgamation of PRP therapy with hydroquinone - a conventional melasma treatment. The results were revelatory, with the combined treatment group witnessing a considerable elevation in their MASI scores and treatment responses. The conspicuous disparity in outcomes, especially in the 51% to 75% improvement bracket between the combined PRP + hydroquinone group and the hydroquinone-only group, underpins the augmented efficacy of the combined regimen.

Mumtaz et al. 2021 [18] investigated the combined effectiveness of PRP therapy and hydroquinone in treating melasma. Surprisingly, the study found that the majority (53.3%) of participants treated with both PRP and hydroquinone saw a 25% to 50% improvement in their MASI scores. In contrast, a higher percentage (76.7%) of participants treated solely with hydroquinone experienced a similar range of improvement. However, the combination of PRP and hydroquinone showed a distinct advantage, with 40% of its users observing a 51% to 75% improvement, compared to only 3.3% in the hydroquinone-only group. Moreover, patient satisfaction scores reflected this trend, with 53.3% of combined treatment users reporting a “good response” versus 27% in the hydroquinone group. This underscores the potential of PRP to amplify the effects of established treatments like hydroquinone.

In a similar vein, Gamea et al. 2022 [19] set out to explore the efficacy of two treatment approaches: the standalone application of a 5% topical TXA in a liposome base and its combination with intradermal PRP injections. Both methods demonstrated positive outcomes. However, patients treated with the combination of topical TXA and PRP (group B) showcased a significantly better response, as substantiated by their satisfaction rates. Side effects were minor, with PRP-related ones being easily manageable and TXA proving well-tolerated among participants. These findings advocate for the synergetic potential of combining 5% topical TXA with PRP injections for enhanced melasma management.

Zhang et al. (2022) [20] focused on a unique combination for melasma treatment, PRP in conjunction with oral TXA. Among the 80 patients, half received the combined treatment, while the other half received only TXA. Interestingly, the combination-treated group exhibited a notably higher efficacy rate (90.48%) than the TXA-alone group (73.68%). The study also tracked serum markers (vascular endothelial growth factor (VEGF), endothelin-1 (ET-1), melanocyte-stimulating hormone (MSH)) and found that while both groups showed changes post-treatment, the alterations in the study group were significantly more pronounced. Furthermore, six months post-treatment, the combined group presented a markedly lower recurrence rate. This research highlights the therapeutic advantage of combining PRP and oral TXA over using TXA alone, especially in ensuring the longevity of the treatment's efficacy.

Lastly, Gharib et al. (2021) [21] contrasted two microneedling adjuncts in melasma treatment: PRP and TXA. The study’s findings indicated a significant reduction in MASI scores during post-treatment sessions in both groups. However, by the study’s end, those treated with microneedling followed by PRP experienced superior results. Notably, 46.15% of patients in the PRP group reported being very satisfied with the results, compared to only 30.77% in the TXA group. The conclusion drawn from this study emphasizes the value of PRP as an effective adjunct to microneedling, suggesting that its integration may offer more satisfactory outcomes in melasma treatment.

## Conclusions

PRP emerges as a promising therapeutic intervention for melasma. Whether as a standalone treatment or combined with treatments like hydroquinone and TXA, PRP presents significant potential in melasma’s clinical management and exhibits remarkable promise in clinical management, these studies collectively elevate the profile of PRP as a potent, innovative, and augmentative treatment for melasma, warranting further expansive trials to substantiate its long-term efficacy and safety.
